# The vascular perspective on acute and chronic lung disease

**DOI:** 10.1172/JCI170502

**Published:** 2023-08-15

**Authors:** Izabela Borek, Anna Birnhuber, Norbert F. Voelkel, Leigh M. Marsh, Grazyna Kwapiszewska

**Affiliations:** 1 Ludwig Boltzmann Institute for Lung Vascular Research, Graz, Austria.; 2 Otto Loewi Research Center, Division of Physiology and Pathophysiology, Medical University of Graz, Graz, Austria.; 3 Pulmonary Medicine Department, University of Amsterdam Medical Centers, Amsterdam, Netherlands.; 4 Department of Pulmonary Medicine, Amsterdam Cardiovascular Sciences, Amsterdam University Medical Centers, Amsterdam, Netherlands.; 5 Institute for Lung Health, German Lung Center (DZL), Cardiopulmonary Institute, Giessen, Germany.

## Abstract

The pulmonary vasculature has been frequently overlooked in acute and chronic lung diseases, such as acute respiratory distress syndrome (ARDS), pulmonary fibrosis (PF), and chronic obstructive pulmonary disease (COPD). The primary emphasis in the management of these parenchymal disorders has largely revolved around the injury and aberrant repair of epithelial cells. However, there is increasing evidence that the vascular endothelium plays an active role in the development of acute and chronic lung diseases. The endothelial cell network in the capillary bed and the arterial and venous vessels provides a metabolically highly active barrier that controls the migration of immune cells, regulates vascular tone and permeability, and participates in the remodeling processes. Phenotypically and functionally altered endothelial cells, and remodeled vessels, can be found in acute and chronic lung diseases, although to different degrees, likely because of disease-specific mechanisms. Since vascular remodeling is associated with pulmonary hypertension, which worsens patient outcomes and survival, it is crucial to understand the underlying vascular alterations. In this Review, we describe the current knowledge regarding the role of the pulmonary vasculature in the development and progression of ARDS, PF, and COPD; we also outline future research directions with the hope of facilitating the development of mechanism-based therapies.

## Introduction

Acute respiratory distress syndrome (ARDS), interstitial lung diseases (ILDs) such as pulmonary fibrosis (PF), and chronic obstructive pulmonary disease (COPD) are all lung diseases that affect gas exchange and limit pulmonary oxygen uptake. Although the alveolar epithelium is often the main research focus, increasing evidence implicates the involvement of endothelial cells (ECs) in lung diseases (1). Under physiological conditions, the metabolically highly active ECs modulate vascular tone, provide barrier integrity, and regulate the entry of immune cells and circulating inflammatory mediators.

Viral infections, lung injury, or exogenous noxious stimuli such as cigarette smoke stimulate ECs to produce vasoconstrictors and pro-remodeling agents as well as cytokines and chemokines (1). These factors modulate the behavior of smooth muscle cells (SMCs) and fibroblasts, and recruit immune cells that might further accelerate disease development.

The progressive occlusion of the vessels by SMC expansion, deposition of extracellular matrix (ECM), and neointima formation can lead to pulmonary hypertension (PH), defined by a mean pulmonary arterial pressure (mPAP) higher than 20 mmHg (2). PH in patients with underlying lung diseases worsens prognosis and survival (3). EC abnormalities and vascular remodeling have been observed in ARDS, diverse forms of ILD including PF, and COPD, although to different degrees, indicating disease-specific mechanisms. Thus, vascular abnormalities might play a more central role in the onset as well as the progression of these lung diseases than previously believed.

This Review focuses on the pathomechanisms of pulmonary vascular remodeling in ARDS and chronic lung diseases (CLDs) such as PF and COPD, which may provide valuable insights into the development of anti-remodeling therapeutic strategies. We will also discuss whether restoration of the vascular components might halt or reverse the parenchymal destruction in lung diseases.

## Pulmonary vascular involvement in ARDS

ARDS can be caused by direct trauma, such as exposure to toxic substances or respiratory infections, as well as by indirect insults like sepsis (4, 5). ARDS is accompanied by severe inflammation and pulmonary vascular dysfunction with diffuse microvascular damage. Pathological changes that develop early in the disease course, e.g., pulmonary vasoconstriction, edema, hypoxemia, embolic vascular obliteration, and capillary damage, can increase pulmonary resistance and cause PH. Studies by Lynne Reid in the 1970s and 1980s showed vascular involvement in patients with ARDS (6, 7). Subsequent studies have since verified the profound vascular damage in ARDS (8–11). As ARDS progresses, there is a loss of capillary density (6) along with SMC hypertrophy and neomuscularization, leading to media thickening (12). However, the long-term effects of ARDS on pulmonary vascular function have not been extensively studied.

Vascular involvement is a common feature of ARDS, particularly in cases of severe COVID-19 (13). Lungs from patients with COVID-19–associated ARDS exhibit pronounced endothelial inflammation including severe endothelial damage, vascular leak, and widespread thrombosis (14). Interestingly, senescent ECs seem to exhibit heightened susceptibility to SARS-CoV-2 infection and subsequent endothelial dysfunction (15). This observation is intriguing, especially since people over 65, who are more likely to have senescent ECs and comorbidities associated with endothelial dysfunction (16, 17), are at higher risk of developing severe COVID-19.

Using single-cell RNA sequencing, multiple lung EC phenotypes have been identified, each potentially playing a unique role in the development of ARDS (18). In the context of virus-induced lung injury, carbonic anhydrase 4–positive (Car4-positive) ECs localize to lung regenerative regions, demonstrating the potential to respond to reparative signals from alveolar type I cells. Furthermore, a highly proliferating EC subpopulation was observed, likely contributing to alveolar revascularization (19). Cumulatively, these findings underscore the multifaceted contributions of ECs in the disease process and reestablishment of lung homeostasis following injury.

### Multifactorial mechanisms of endothelial dysfunction in ARDS

**Figure 1:**
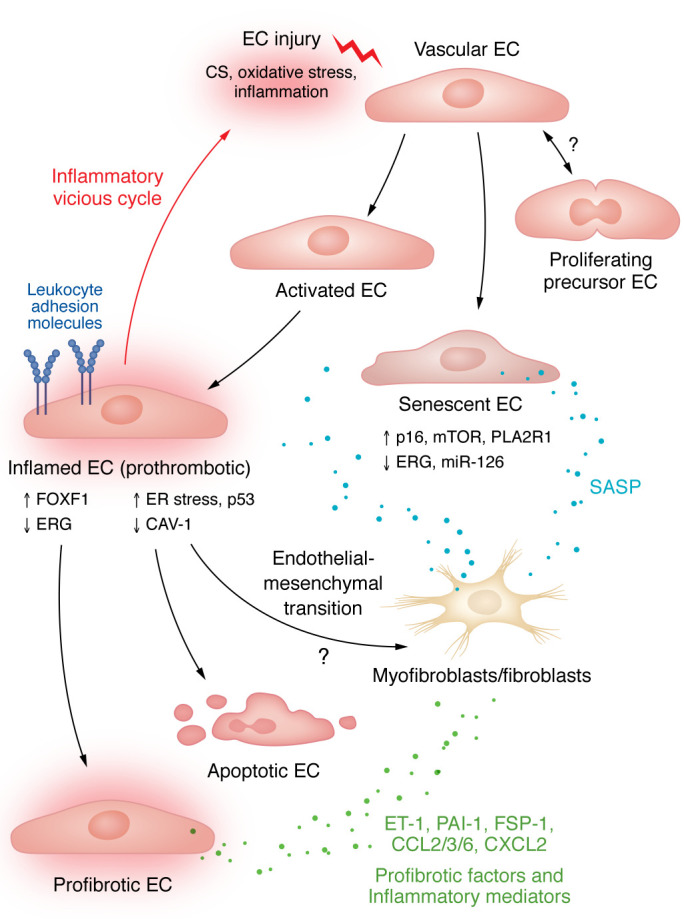
Mechanisms of endothelial dysfunction. Endothelial cell (EC) injury can occur through a variety of stressors, such as cigarette smoke (CS), inflammation, and oxidative stress, leading to an activated and inflamed EC, which may manifest as ER stress and/or p53 dysregulation, or Cav-1 downregulation. Deregulation of transcription factors, such as FOXF1 or ERG, promotes a fibrosis-conducive EC phenotype. Inflamed ECs express a variety of adhesion molecules, further contributing to the recruitment of inflammatory cells. If uncontrolled, aberrant regulation of these processes can lead to a vicious cycle of sustained inflammation and tissue destruction. EC injury can also induce cellular senescence, advancing tissue inflammation, myofibroblast formation, and remodeling via a senescence-associated secretory phenotype (SASP). The extent to which proliferating EC precursors can substitute injured ECs and restore EC homeostasis still needs to be determined.

An ongoing inflammatory response and increased oxidative stress cause endothelial injury leading to vessel permeability. This process is further compounded by the release of interstitial proinflammatory mediators into the bloodstream, which attract additional immune cells to the site of injury and perpetuate the inflammatory cycle (Figure 1). Various mechanisms involved in endothelial dysfunction, including the inflammatory response, the cellular stress response, and the fibroproliferative response, offer potential avenues for future therapeutic investigations.

#### Inflammatory response.

An activated endothelium expresses higher levels of platelet and leukocyte adhesion molecules, including E-selectin, P-selectin, ICAM-1, and VCAM-1 (20, 21), leading to a procoagulant and immunoreactive phenotype and fibrin deposition (22). The interaction of platelets with neutrophils results in reciprocal activation and drives so-called immunothrombosis (23). The procoagulation state in ARDS has been the focus of several therapeutic approaches. However, systemic administration of recombinant human-activated protein C (24), antithrombin (25), and tifacogin (tissue factor pathway inhibitor; ref. 25) all yielded disappointing clinical outcomes, proving modulation of disturbed coagulation in patients with ARDS to be a challenging target.

During the inflammatory response, neutrophils are recruited to the site of injury, where they release neutrophil elastase (NE). NE proteolytic activity has been shown to damage protective, endothelial glycocalyx (26) and to degrade ECM proteins, releasing growth factors bound to the ECM, and activating other proteases and inflammatory mediators, thereby amplifying the inflammatory response and tissue damage (27). Considering the central role of neutrophil-mediated vascular injury in the development of vascular dysfunction, inhibiting the proteolytic activity of NE could provide a plausible strategy for preserving vascular integrity. Indeed, clinical trials regarding the use of NE inhibitor in treating ARDS are currently being conducted (Table 1).

Another important regulator of pulmonary vascular homeostasis is caveolin-1 (Cav-1), which has been implicated in ARDS and CLDs. This scaffolding protein plays a crucial role in structural support and cellular signaling, contributing to the overall integrity and functionality of the pulmonary vasculature (28). In ARDS lung tissue, a noticeable reduction in Cav-1 expression occurs specifically in the endothelial layer of remodeled vessels (11). *Cav1^–/–^* mice develop pronounced lung abnormalities, including hypercellularity, alveolar wall thickening, extensive pulmonary vascular remodeling, and PH (29, 30). These observations suggest that the deregulation of Cav-1 might be an important initiating factor in EC dysfunction, ultimately leading to vascular remodeling.

#### Cellular stress response.

In ARDS, various stressors cause endoplasmic reticulum (ER) stress, which is central to endothelial dysfunction. The unfolded protein response (UPR) helps to maintain protein homeostasis in the ER (31). Activation of UPR in pulmonary endothelium increases levels of the tumor suppressor protein p53 (32), leading to inhibition of the inflammatory RhoA pathway, activation of Rac1, and ultimately enhanced vascular barrier function (33). p53 is crucial for maintaining vascular integrity, and its induction counteracts endothelial hyperpermeability and suppresses inflammatory NF-κB signaling (32, 34). Several therapeutic avenues that inhibit ER stress also exert protective effects in vascular endothelium by augmenting p53 and activating UPR branches; and inhibitors of ER stress emerge as therapeutic targets to prevent ARDS endothelial dysfunction. Despite different modes of action, heat shock protein 90 (HSP90) inhibitors (35–37) and growth hormone–releasing hormone (GHRH) antagonists (38, 39) induce p53 and trigger UPR, thus counteracting endothelial hyperpermeability (40). The promising results of preclinical studies suggest that UPR-modulating agents could provide therapeutic benefits for patients with ARDS by strengthening the vascular barrier.

The current understanding of the cellular stress response highlights the complexity of the underlying mechanisms involved in lung injury, emphasizing the need for a holistic approach that encompasses the intricate interplay between ECs, other cellular components, and the various factors they produce. Therefore, the development of treatment strategies necessitates a consideration of the vasculature’s role in the pathological process as well as the sequence of cell-cell interactions, since distinct factors come into play at different stages of the disease.

#### Fibroproliferative responses.

ARDS pathobiology entails a transition from inflammation to fibrosis (41) characterized by increased collagen production (42) and excessive deposition of ECM components (43). Regardless of the causal agent, patients with ARDS face a heightened risk of developing lung fibrosis due to sustained damage to the lung parenchyma and vasculature (44–46). Endothelial dysfunction plays a crucial role in disturbing the delicate equilibrium between profibrotic and antifibrotic signals (Figure 1). In the bleomycin-induced injury model, the deletion of the endothelial transcription factor forkhead box F1 (FOXF1) leads to decreased expression of Ras-related protein (R-Ras), a vital regulator of EC barrier function and repair (47, 48). This process results in a fibrosis-conducive EC phenotype (46). Persistent EC activation causes the release of profibrotic factors (e.g., plasminogen activator inhibitor-1 and fibroblast-specific protein-1) and inflammatory mediators (CCL2, CCL3, CCL6, and CXCL2), creating an environment conducive to fibrosis development (49–53).

## Pulmonary vascular remodeling in chronic lung diseases

CLDs include COPD and ILDs such as idiopathic PF (IPF) and systemic sclerosis–associated ILD (SSc-ILD). Independent of disease entity, the presence of PH in CLDs (termed “group 3 pulmonary hypertension associated with lung diseases and/or hypoxia”; ref. 2) is associated with reduced exercise capacity, greater need for oxygen supplementation, decreased quality of life (54–56), and, in patients with severe PH, a worse prognosis (57, 58). Despite extensive research efforts, PH in CLDs continues to represent an unmet medical need.

Some clinicians posit that vascular remodeling and PH in CLDs are a consequence of hypoxia in areas of insufficient ventilation and diffusion (59). However, studies in several mouse models have shown that vascular remodeling can occur independently of hypoxia and precede the development of bleomycin-induced PF, Fra-2–induced SSc-ILD, and tobacco smoke–induced emphysema (60–63), suggesting that vascular alterations might play a more active role in the progression of underlying lung diseases than previously believed.

CLDs are age associated, and indeed, age-related changes, such as genomic instability, epigenetic alterations, and cellular senescence, have been described as affecting and contributing to the development of CLDs (64, 65). In addition to natural aging, cellular senescence can be induced by oxidative stress or smoking. Although senescent cells exhibit an arrested cell cycle, they remain metabolically active and can promote chronic inflammation via senescence-associated secretory phenotype (SASP), which is characterized by the release of inflammatory mediators and growth factors (66). It is conceivable that a so-called spillover of inflammatory factors from the lungs into circulation may affect the vascular system.

### Vascular remodeling in interstitial lung disease

ILDs are characterized by progressive scarring and fibrosis of the lung, due to aberrant ECM deposition and proliferation of α-SMA–positive and –negative fibroblasts (67, 68). Although the progressive fibrotic nature of these diseases is a common denominator, the vasculature is also altered. In line with this, ILD lungs contain high numbers of severely remodeled vessels and exhibit mild to moderate PH (69).

While ECs were believed to be innocent bystanders, they have increasingly gained recognition as active drivers of ILDs (70) (Figure 1). Fibrotic lung regions show fewer ECs (71). Furthermore, in IPF there are fewer general capillary ECs capable of functioning as progenitors during homeostatic maintenance or repair after lung injury (72), suggesting that diminished regenerative properties of ECs contribute to persisting fibrosis (73).

#### Endothelial perturbations.

The central role of ECs in the development and progression of vascular remodeling in lung fibrosis has been highlighted by a plethora of current studies. Endothelium-specific knockout of endothelin-1, a potent vasoconstrictor and profibrotic factor involved in PF (74) and PH (75), ameliorates vascular remodeling in the bleomycin mouse model, without affecting PF (76). In a TGF-β1–overexpressing rat model and in patients with PH and PF (PH-PF), EC dysfunction and vascular remodeling are associated with enhanced fibrosis (77). The contribution of ECs to pulmonary homeostasis and repair has been documented by EC-specific deletion of hepatocyte growth factor (HGF), which actively contributes to NOX4 activation in perivascular fibroblasts during bleomycin- or acid-induced lung injury in mice (78). Similarly, the presence of the endothelial transcription factor ETS-related gene (ERG) within the capillary endothelium is a prerequisite for tissue repair, and loss of ERG impairs the resolution of fibrosis following bleomycin-induced lung injury (79).

The endothelium not only contributes to tissue remodeling through endothelial-parenchymal crosstalk, but also serves as a recruitment site for circulating progenitor cells. For example, circulating endothelial colony-forming cells (ECFCs), which expand clonally and have the ability to form new blood vessels in vivo (80), have gained attention in vascular remodeling. ECFCs might contribute to IPF pathogenesis via higher prothrombotic potential (81), or through an apoptotic, senescent, and IL-8–producing phenotype that promotes neutrophil infiltration in vitro (82). Indeed, elevated levels of ECFCs with increased proliferative potential correlate with worse gas exchange in IPF (83) and diminished lung function in SSc-ILD (84). However, the role of ECFCs in PF has been questioned, as injection of ECFCs (from cord blood or IPF patient blood) failed to influence disease outcome in the bleomycin mouse model (85). Taking into account the lack of clear definition and ambiguities in the field of ECFCs (86), much more work is needed to understand their potential role in vascular remodeling in ILDs.

#### Endothelial-immune interface.

Activated ECs express a variety of adhesion molecules (such as E-/P-selectin and ICAM-1/VCAM-1), which can also be found in the circulation of patients with PF/ILD and often correlate with poor lung function and outcome (70). These factors also crucially contribute to the recruitment and extravasation of inflammatory cells. In pulmonary arteries of fibrotic lungs, changes in T cells, macrophages, myeloid-derived suppressor cells (MDSCs), and mast cells have been reported and linked to vascular remodeling (87–89). Increased levels of lung MDSCs worsened vascular remodeling and PH without affecting parenchymal fibrosis in a mouse model of bleomycin-induced lung fibrosis (90, 91). Also, macrophages play a crucial role in PH development in the setting of PF. In the rat bleomycin-monocrotaline model, the inhibition of Slug (encoded by *SNAI2*), which is highly expressed in macrophages in PH-PF lungs, ameliorates PH (92). Furthermore, reducing macrophage infiltration via blockade of macrophage migration inhibitory factor (MIF) attenuated PH in mice following bleomycin administration (93, 94). In contrast to the increased macrophage numbers, natural killer T (NKT) cells were decreased in vessels of patients with PH-PF, accompanied by increased collagen (88). Restoration of NKT cells decreased vascular as well as parenchymal remodeling and PH in the bleomycin-induced fibrosis mouse model and reduced collagen deposition via activation of the STAT1/CXCL9/CXCR3 axis (88).

Chemokines and cytokines are important messengers in endothelial-inflammatory communication, although their effects can vary depending on the cell type involved. For instance, in the bleomycin mouse model, the knockout of *CXCR2* in MDSCs ameliorated vascular remodeling and PH; however, EC-specific *CXCR2* knockout worsened outcomes due to increased numbers of MDSCs (91). Additionally, IL-11 and its receptor IL-11Rα showed increased expression in pulmonary arterial SMCs (PASMCs) and ECs of IPF patients with PH versus those without PH (95). Exogenous administration of IL-11 in mice was sufficient to induce vascular remodeling, PH, and PF (95), highlighting the role of this cytokine in the development of PF-associated vascular remodeling.

Activated T cells represent another potent source of cytokines. Th2 inflammation, in particular, is a known driver of pulmonary remodeling, especially in systemic sclerosis (SSc). Many invaluable insights into the pathological processes involved in vascular remodeling have been gained from the SSc-ILD mouse model that is induced by Fra-2 overexpression. This model is characterized by progressive vascular remodeling, PF, chronic Th2 inflammation, and a concomitant decrease in regulatory T cells (Tregs) (62, 96, 97). Importantly, it was shown that Th2 cytokines, such as IL-4, predispose the ECs to exacerbated injury, leading to an aggravated disease phenotype following treatment with the antifibrotic drug pirfenidone in Fra-2–overexpressing mice, but not in bleomycin-treated mice (98). The clinical importance of restoring T cell homeostasis was also highlighted by treatments that improve vascular remodeling in this model, including the T cell costimulation blocker abatacept (99) and restoration of Tregs by either adoptive transfer or low-dose IL-2 treatment (100). Collectively, these studies emphasize the therapeutic potential of restoring EC-immune homeostasis. Furthermore, they provide valuable insights into the importance of understanding underlying disease pathomechanisms, particularly the specific inflammatory conditions that trigger preactivated endothelium and potentially lead to unfavorable outcomes.

#### Oxidative stress and ECM turnover.

Inflammation is associated with oxidative stress that can induce cellular senescence, further promoting vascular remodeling. Mice lacking antioxidant extracellular superoxide dismutase 3 (SOD3) display worsened silica-induced PH-PF, with vascular remodeling in all pulmonary areas, while vascular remodeling in WT mice was limited to fibrotic areas (101). Many proteins involved in tissue homeostasis and repair, such as SOD3 or ERG, are decreased in older adults (79, 102). Taking into account our aging population and the higher prevalence of PF among older individuals, understanding these pathways becomes crucial in addressing the emerging health care challenge.

Oxidative stress is associated with DNA damage. Increased levels of the checkpoint inhibitors CHK1 and CHK2, members of the DNA damage control and repair system, have been reported in patients and animal models of PH-PF (103) (Table 2). CHK1/2 inhibition improves vascular remodeling and hemodynamics by preventing fibroblast-to-myofibroblast transition (103). The endothelial transcription factor sterol regulatory element–binding protein 2 (SREBP2), which is linked to oxidative stress response, contributes to vascular remodeling. SREBP2 is highly expressed in PF lungs and supports mesenchymal properties, thereby aggravating vascular remodeling (51). Also, dysregulation of protein translation via decreased eukaryotic translation initiation factor 2 alpha kinase 4 (EIF2AK4) expression was documented in patients with PH-PF. Mutations of EIF2AK4 further worsened PH-PF in a rat bleomycin model, suggesting causative relations (104).

The ECM and its associated proteins influence cellular behavior and contribute to vascular remodeling; one example is the matricellular CCN family of intercellular signaling proteins. In bleomycin-treated mice, CCN2, also known as connective tissue growth factor, was partially responsible for PH development (105), while CCN3 had beneficial effects on lung endothelial homeostasis, partially by antagonizing CCN2 expression (106). Galectin-3, a known profibrotic lectin, was also suggested to induce endothelial-mesenchymal transition in bleomycin-induced PF (107) and is currently the focus of a clinical trial in patients with IPF (108). Furthermore, hyaluronan, a major component of the ECM, and hyaluronan synthase 3 (HAS3) were upregulated in response to adenosine signaling and contributed to vascular remodeling in PF (109). Inhibition of this pathway using adenosine depletion or 4-methylumbelliferone, a blocker of HAS3 activity, ameliorated vascular manifestations in the *Ada^–/–^* model of PH with combined pulmonary fibrosis and emphysema (CPFE) (109). 4-Methylumbelliferone also alleviated vascular pathologies in Fra-2 overexpression and graft-versus-host disease mouse models of SSc-ILD (110), further corroborating the important role of the ECM and/or hyaluronan production in the development of vascular remodeling in PF. Indeed, 4-methylumbelliferone is currently under investigation for the treatment of PH-ILD in a phase II clinical trial (ClinicalTrials.gov NCT05128929).

These studies underscore the central role of ECs in injury and repair processes, connecting immune cell homeostasis, cellular senescence, oxidative stress, and ECM deposition in the development of vascular remodeling and lung fibrosis (Table 2). In the future, our focus should shift toward better understanding the interplay between these processes and disease dynamics. Instead of seeking and treating a singular culprit, the objective should be to restore tissue homeostasis, thereby improving the overall disease phenotype. Furthermore, it has to be kept in mind that different ILD entities with diverse underlying pathomechanisms may require specific treatments, as pre-injured ECs could be further activated, thereby initiating detrimental rather than beneficial effects.

### Vascular remodeling in COPD

COPD is the most common CLD and the third most common cause of death worldwide (111). It is characterized by the destruction of parenchyma, airspace enlargement, and airway remodeling with aberrant mucus production and hyperresponsiveness (112). In COPD, PH is typically mild to moderate; however, up to 4% of COPD patients present with severe PH but only mild to moderate airway obstruction (113, 114). Patients in this group are described as having the pulmonary vascular phenotype (115). Pulmonary vascular alterations in these patients include medial hypertrophy and intimal thickening, with the degree of remodeling depending on disease severity (116, 117). In COPD accompanied by severe PH, vascular remodeling is more morphologically similar to that seen in idiopathic pulmonary arterial hypertension (PAH) than to that noted in COPD with mild to moderate PH (117, 118). However, vascular remodeling can also be observed in explanted COPD lungs even without PH (116, 119).

The pathological mechanisms underlying the development of PH in COPD (PH-COPD) are still poorly understood. Early triggers may include endothelial dysfunction, especially in combination with cigarette smoke exposure, leading to inflammatory and oxidative stress. Hypoxia exposure additionally damages ECs, and leads to vasoconstriction and consequently compensatory remodeling (120, 121). It has also been postulated that progressive capillary loss leads to the simultaneous loss of terminal bronchioles and associated arteries (122–124).

The loss of endothelium is an early event in emphysema development, where changes in VEGF signaling promote cell apoptosis (125). Furthermore, isolated pulmonary arteries from COPD samples exhibit endothelial dysfunction (126, 127). Although advanced COPD does not associate with EC population shifts (neither in the macro- nor the microvasculature), EC gene expression profiles indicate an increase in the inflammatory signaling stress response and a decrease in vessel development (128). In an elastase-induced emphysema mouse model, disruption of the pulmonary endothelium promotes a pro-angiogenic state, and i.v. injection of healthy lung ECs reversed emphysema (129). Many factors released during EC activation and injury recruit and modulate immune cells, which might influence and further accelerate the tissue pathobiology.

#### Cellular senescence.

One of the most important risk factors for COPD is old age. Age-related processes, including cellular senescence, indeed, crucially contribute to the pathomechanisms of COPD. For example, in patients with COPD, expression of phospholipase A_2_ receptor 1 (PLA2R1) is increased and localized to alveolar epithelial type II cells, ECs, and PASMCs. In mice, overexpression of PLA2R1 induces EC senescence, lung emphysema, and PH (130). The senescence-associated mTOR pathway is activated in COPD lungs and drives EC senescence and emphysema (131). In addition, several microRNAs have been implicated in the regulation of cellular senescence or vascular remodeling in COPD (132–136) (Table 3). Exemplarily, microRNA-126 (miR-126), which has well-documented roles in lung regeneration and homeostasis, is downregulated in senescent ECs (137) and was recently linked to vascular remodeling in COPD (138) (Figure 1).

#### Oxidative stress.

Independent of age, oxidative stress can induce cellular senescence. Increased oxidative stress in COPD can derive from structural and immune cells. Recent evidence has suggested a role for macrophage iNOS (NOS2) in mediating smoke-induced PH (139). iNOS, an enzyme involved in the macrophage inflammatory response and upregulated by hypoxia or proinflammatory cytokines, such as tumor necrosis factor-α (TNF-α), interleukin 6 (IL-6), or interferon-γ (IFN-γ), plays a crucial role in the development of tobacco smoke–induced emphysema and PH in mice (61). However, specific deletion of iNOS in the bone marrow or macrophages protects against smoke-induced PH, but not emphysema (61, 139). Similarly, ROS-induced activation of the non-lysosomal cysteine protease calpain contributes to vascular but not parenchymal remodeling in COPD (140). Neuronal nitric oxide synthase 1 (NOS1) was recently revealed to be a direct target of miR-4640-5p, whose expression is markedly higher in PH-COPD lung tissue compared with healthy controls (136). NOXO1, a subunit of the non-phagocytic NADPH oxidase, is a source of superoxide, which drives emphysema and PH in cigarette smoke–exposed mice (141, 142). The expression of another NADPH subunit, NOX4, correlates with increased pulmonary vascular wall volume in COPD lungs (143), where it is speculated to promote ROS production and distal pulmonary vascular remodeling. Interestingly, high levels of the antioxidant SOD3 can also contribute to vascular remodeling in COPD, through increased levels of hydrogen peroxide (144). Cumulatively, these studies highlight the contribution of oxidative stress to vascular remodeling and shed light on its potential as a therapeutic target. Indeed, antioxidant treatment using MitoQ, which targets mitochondrial ROS production, restored endothelial barrier function and diminished activation of proinflammatory pathways in ECs (145).

#### Immune cell alterations.

In the pulmonary arterial wall of patients with COPD, an increase in the number of CD45^+^ cells has been observed, along with a decrease in the number of circulating progenitor cells. These changes were associated with endothelial dysfunction and vessel remodeling (146). In the adventitial layer, total leukocytes increase, especially CD8^+^ T cells, albeit with no changes in neutrophil or macrophage numbers. Furthermore, the total number of leukocytes was associated with the degree of intimal thickening (147). Although the number of monocytes and macrophages in COPD vessels is maintained, these cells may still contribute to vascular remodeling in PH-COPD. In emphysema, the arginine methyltransferase PRMT7 promotes the extravasation of monocytes, resulting in tissue injury (148). Similar mechanisms may also damage the vascular wall and contribute to vascular remodeling. COPD lungs possess increased tertiary lymphoid structures, which are rich in B and T cells. As tertiary lymphoid structures have been linked with idiopathic PAH (149), it would be useful to determine their relevance in PH-COPD. In line with this, regulatory B cells (Bregs) were downregulated in the circulation of COPD patients, and their ability to produce IL-10 in response to cigarette smoke exposure was limited, contributing to the inflammatory milieu in COPD lungs (150). In addition, Bregs are involved in COPD vascular remodeling by influencing T cell differentiation (toward Th cells and away from Tregs) and PASMC proliferation (151).

PH-COPD is also associated with increased circulating cytokines including IL-6 (152), TNF-α (153), and the alarmin HMGB1 (154), all of which are strongly implicated in PH pathogenesis. Increased IL-6 plasma levels correlate with mean pulmonary arterial pressure (mPAP), further supporting the role of inflammation in the pathogenesis of PH-COPD (155). TNF-α is a potent activator of ECs, facilitating inflammatory cell recruitment. HMGB1 acts as a chemoattractant and induces the proliferation of PASMCs and ECs, via ERK/JNK and AP1 (153).

Vascular remodeling in COPD remains an enigma, and much work is still needed to delineate its active contribution to COPD development. Again, ECs appear to play a dominant role, as a multitude of factors contribute to their dysfunction, ultimately leading to vascular remodeling and emphysema (Table 3). Further investigations are warranted to comprehensively explore the impact of oxidative stress, cellular senescence, and the immune system, not only within the local vasculature and lungs but also on a systemic level.

### Conclusions, future challenges, and opportunities

**Figure 2:**
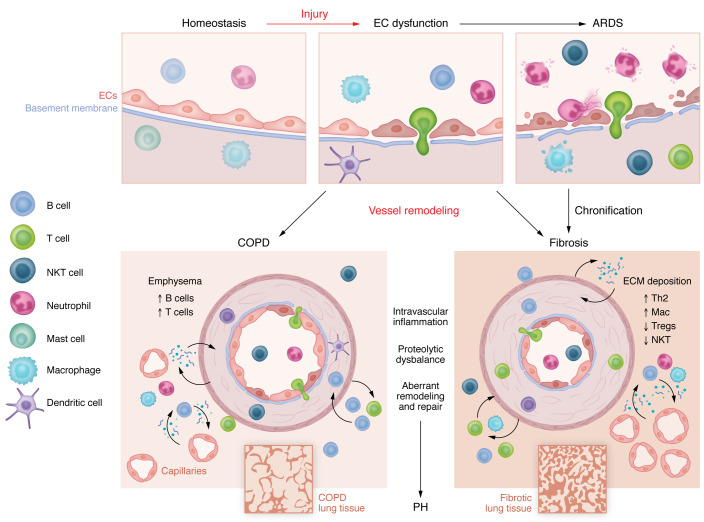
Endothelium at the center of tissue maintenance and development of CLDs. The endothelium plays a key role in regulating vessel homeostasis by exerting a predominantly inhibitory effect on inflammation. Different stressors can activate ECs and induce loss of their barrier function, promoting leukocyte adhesion and transendothelial migration. Exacerbated EC dysfunction together with activation of inflammatory response and recruitment of leukocytes creates an inflammatory storm, leading to tissue destruction and subsequent remodeling. If not resolved, or if sustained tissue damage is too great, this process can lead to the onset of progressive fibrosis in the lung tissue. However, beyond acute EC damage, chronic EC dysfunction also emerges as an important player in CLD development. Vascular alterations — although to a different degree — are commonly found in CLDs, irrespective of their etiology, and can lead to the development of PH. Importantly, vessels contribute to the regulation of immune cell homeostasis and actively participate in the propagation of tissue damage, as seen in COPD, or remodeling, as occurs in PH-PF.

**Figure 3:**
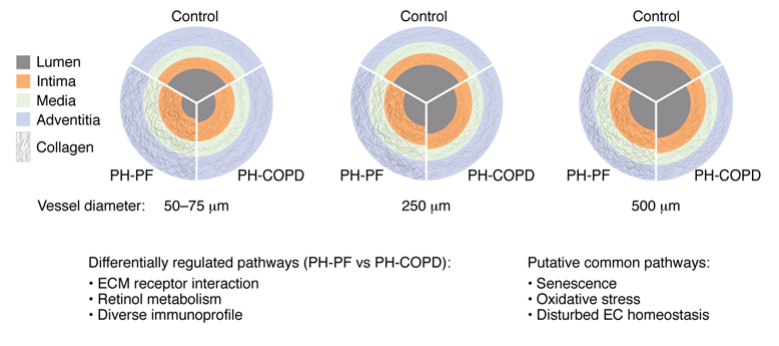
Vascular remodeling in the lungs of PH-PF and PH-COPD patients. Pulmonary arteries of patients with PH-PF present with more pronounced intimal and medial remodeling, reflected as thickness changes that vary with vessel size. The vessels of patients with PH-PF also show increased collagen deposition as compared with those from patients with PH-COPD. The two conditions are further distinguished by diversity in corresponding immune cell profiles (116, 156, 157). Differential gene expression and regulated pathways, including those related to the ECM and retinol metabolism, underscore the differences between PH-PF and PH-COPD (116). However, several general mechanisms, such as cellular senescence, oxidative stress, and disturbed EC homeostasis, seem to be shared in the vascular remodeling processes of patients with PH-PF and PH-COPD.

Despite the divergent effects of ILD and COPD on the lung parenchyma, with ILD associated with abnormal fibrosis and COPD characterized by tissue degradation, both diseases share the common complication of vascular remodeling, which worsens patient outcomes. Pulmonary vascular remodeling in PH-PF is particularly notable, marked by substantial collagen deposition, whereas in PH-COPD, it is comparatively less pronounced (116, 156, 157) (Figures 2 and 3). The diverse vascular alterations are reflected by different gene expression patterns and immune cell composition in pulmonary arteries isolated from these two entities (116, 157), indicating that targeted treatments for each disease may be necessary. Despite these differences, several pathways, such as oxidative stress and cellular senescence, seem to be shared in the vascular remodeling of ILDs and COPD (Figure 3). However, our understanding of common and diverse underlying pathomechanisms remains limited by a lack of comparative studies. Furthermore, mechanistic and functional studies often rely on animal models, which despite their advantages often do not fully recapitulate human disease (Table 4). Especially in complex diseases such as ILDs or COPD, characterized by diverse etiologies and/or endotypes, animal models have to be chosen with care depending on the underlying pathomechanism to fully exploit their translational potential.

Substantial progress has been made in characterizing pathomechanisms driving vascular remodeling in various CLDs. However, it is important to acknowledge that our current understanding of the disturbed EC function and immune homeostasis remains limited and represents only the tip of the iceberg. Delineating interconnections and crosstalk between the endothelium, surrounding structural cells, and the immune system will help in understanding disease pathobiology. Here, single-cell omics approaches can give excellent insights into intercellular communication and interaction pathways. In addition, artificial intelligence and machine learning will provide unknown opportunities with respect to image analysis and multi-omics data interpretation. However, confirmatory approaches and in vivo studies will be needed to validate the postulated interdependencies. The next big challenge will be to target cell type–specific alterations delineated by these technologies. Nevertheless, a better understanding of the molecular and cellular processes underlying vascular pathology could pave the way for the development of targeted interventions that can effectively restore tissue homeostasis and improve clinical outcomes in a disease-specific manner.

## Therapeutic targeting of vascular remodeling in lung diseases

### Targeting the vasculature in ARDS

Similarities in the mechanism of vascular remodeling between PAH and ARDS have led to the investigation of PAH drugs as potential treatments for ARDS (Table 1). However, despite promising results from animal studies, these positive outcomes have not yet translated into clinical benefits for patients. Inhaled pulmonary vasodilators, despite their physiological advantages, such as improving ventilation/perfusion mismatch and reducing hypoxia-induced vasoconstriction and PH, did not decrease overall mortality (158, 159). Interestingly, the effectiveness of interventions appears to be influenced by the timing of their implementation. For example, the use of epoprostenol, a synthetic prostacyclin, was found to worsen oxygenation when administered to ARDS patients with pneumonia. However, when administered during secondary ARDS, epoprostenol improved oxygenation (160).

Numerous other pharmacological interventions have been tested in ARDS patients. However, none provided notable clinical benefits. As selective pharmacological interventions have not yielded the anticipated therapeutic benefits, it may be necessary to shift toward an approach that takes into account the disease dynamics and multitude of factors contributing to the syndrome’s mechanisms.

### Targeting the vasculature in ILDs

Several clinical trials assessing the efficacy of endothelin receptor blockers, such as ambrisentan, bosentan, and macitentan, in treating patients with IPF did not show clinical benefits (Table 1). Notably, the trial involving ambrisentan had to be prematurely terminated because of safety concerns (161–164). Similarly, a clinical study with riociguat in idiopathic interstitial pneumonia–associated PH was discontinued due to an increased risk of hospitalization and death (165). Also, sildenafil did not meet the primary endpoint of improving the 6-minute walk distance (6MWD) in patients with IPF, although small improvements in secondary endpoints, including the degree of dyspnea and quality of life, were observed (166). Trials investigating sildenafil in combination with antifibrotic treatment such as nintedanib (167) or pirfenidone (168) did not provide added clinical benefit for ILD patients.

However, the recent INCREASE trial provided compelling evidence supporting the use of inhaled treprostinil in the treatment of PH in ILD (PH-ILD). In comparison with patients receiving treatment solely for the underlying lung disease, the addition of treprostinil improved 6MWD and N-terminal pro–B-type natriuretic peptide (NT-proBNP) levels and decreased the risk of clinical worsening (55). Furthermore, post hoc analysis showed that treprostinil inhalation had a positive influence on forced vital capacity (169).

### Targeting the vasculature in COPD

In the context of PH-COPD, the administration of PAH-specific therapy also has shown limited benefits (Table 1). The available evidence from clinical studies with bosentan remains inconclusive. On one hand, administration of bosentan in COPD patients with Global Initiative for Chronic Obstructive Lung Disease (GOLD) grading stages III–IV (severe and very severe) did not lead to an improvement in the 6MWD and worsened hypoxemia due to ventilation/perfusion mismatch (170). On the other hand, a small preliminary study involving patients with PH-COPD reported positive outcomes, including improvements in mPAP, pulmonary vascular resistance (PVR), and 6MWD (171). Similarly, the investigation of sildenafil in PH-COPD has yielded mixed results. One clinical trial showed that sildenafil treatment did not improve 6MWD or oxygenation (172). However, another study found that sildenafil administration decreased PVR, and improved the body mass, airflow obstruction, dyspnea, exercise capacity (BODE) index and diffusion capacity of the lung for carbon monoxide (DLCO) (173). Currently, it is proposed that patients with PH-COPD who present with pulmonary vascular phenotype might benefit from vasoactive medications (115).

### Future treatment options and considerations

Despite the numerous clinical trials conducted in PH related to CLD, the practical impact on patient outcomes remains limited (Table 1). A meta-analysis of studies investigating PAH-specific therapies in PH occurring in CLDs has concluded that there is insufficient evidence to justify the routine use of PAH-specific therapy in the context of CLD (134). It is evident that vasodilators offer limited clinical benefits, highlighting the need for a more holistic approach that takes into account the underlying pathomechanisms of lung disease. For instance, inhaled treprostinil is effective, and FDA approved, for the treatment of PH-ILD, but lacks sufficient evidence for use in PH-COPD, indicating that the underlying lung disease can lead to different patient outcomes. The positive effects of inhaled treprostinil in ILD might result from direct improvements in the vascular compartment that occur as a consequence of inhibition of parenchymal fibroblast proliferation or from modulation of the local inflammatory milleu (174, 175). Targeting proliferative mechanisms in the vasculature and parenchyma appears to benefit patients with PH-ILD; however, this strategy might be deleterious in PH-COPD, in which the lung parenchyma is mostly destroyed. Here, restoration of regenerative pathways, rather than antiproliferative approaches, will be needed to successfully treat PH-COPD without worsening the underlying disease.

Mounting evidence supports the crucial role of the vasculature in acute lung disease and CLDs. Notably, CLDs are diseases of the older population in which dysfunctional endothelium plays a key role. Also, the mortality of ARDS patients increases with age, possibly as a result of so-called “exhausted” ECs. Therefore, addressing EC senescence and dysfunction becomes a vital step in reestablishing tissue homeostasis. Since immune cells and EC function are closely interconnected, the development of therapeutic strategies aimed at supporting endothelial integrity and function must also address the inflammatory aspects.

## Perspectives and outlook

Although our knowledge is expanding, a holistic view of acute and chronic lung diseases has been confounded by a compartmentalized view of these diseases. Past oversights regarding the pathological contribution of the vasculature in the setting of ILDs or COPD may have hindered the development of successful treatment strategies. By bringing together all the results gained in recent years, it becomes clear that lung diseases cannot be defined purely as airway disease or interstitial disease, as pathobiology involves the entire lung. In addition, the participation of the systemic circulation and the bone marrow as supply lines for recruited inflammatory and immune cells should not be ignored. Consideration for the vasculature in ARDS and CLDs will add additional complexity but also expand therapeutic possibilities in targeting intravascular inflammation and the endothelial–immune cell interaction. It is also plausible that rebalancing oxidative stress and senescence might prolong the longevity of ECs, thus preventing the development of CLDs. Furthermore, gaining a comprehensive understanding of diverse EC phenotypes, and characterizing the trajectories of disease progressions, will be instrumental in delineating the pivotal transition of ECs from a reparative role to pathobiological involvement. It is still unclear whether it will be possible to restore EC homeostasis; however, given the key role of ECs in acute and chronic lung diseases, this lofty goal is definitely worth seeking. We believe that changing our compartment-oriented view of lung diseases is a prerequisite for the successful development of therapies that restore perfusion and gas exchange and reestablish immune, endothelial, and tissue homeostasis to improve lung function and, ultimately, the survival of patients with lung diseases.

## Author contributions

IB and AB made equal contributions to the literature search and manuscript preparation. IB additionally performed final proofreading, editing, and adjustments, which is why she is listed first.

## Figures and Tables

**Table 1:**
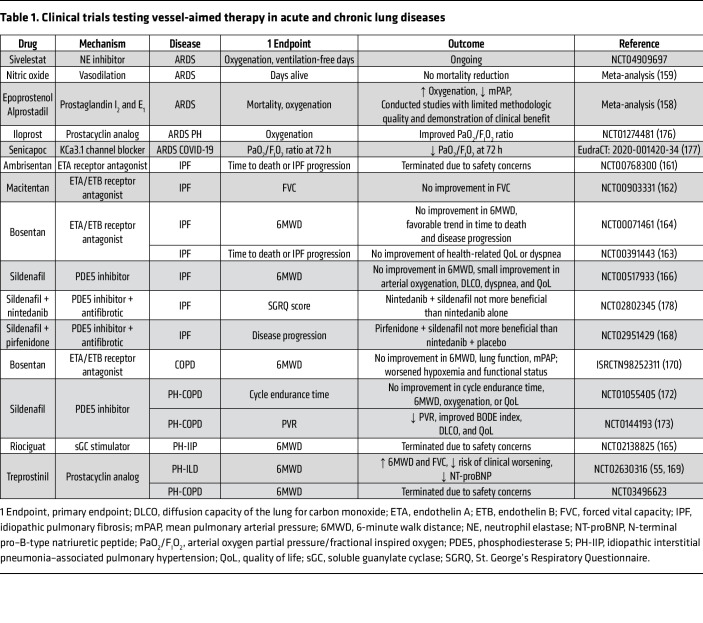
Clinical trials testing vessel-aimed therapy in acute and chronic lung diseases

**Table 2:**
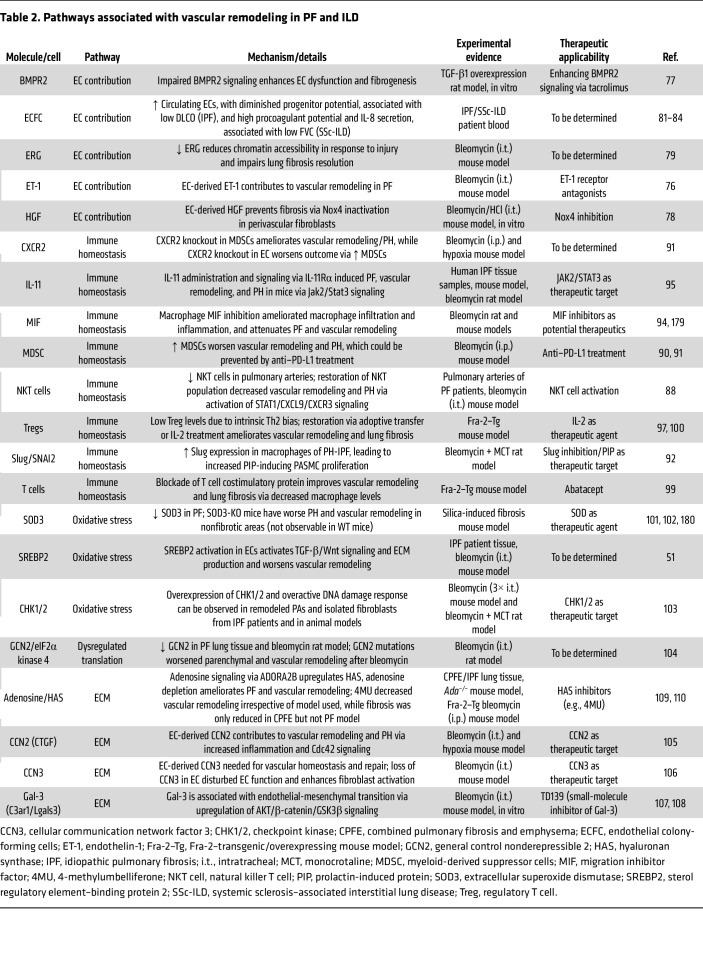
Pathways associated with vascular remodeling in PF and ILD

**Table 3:**
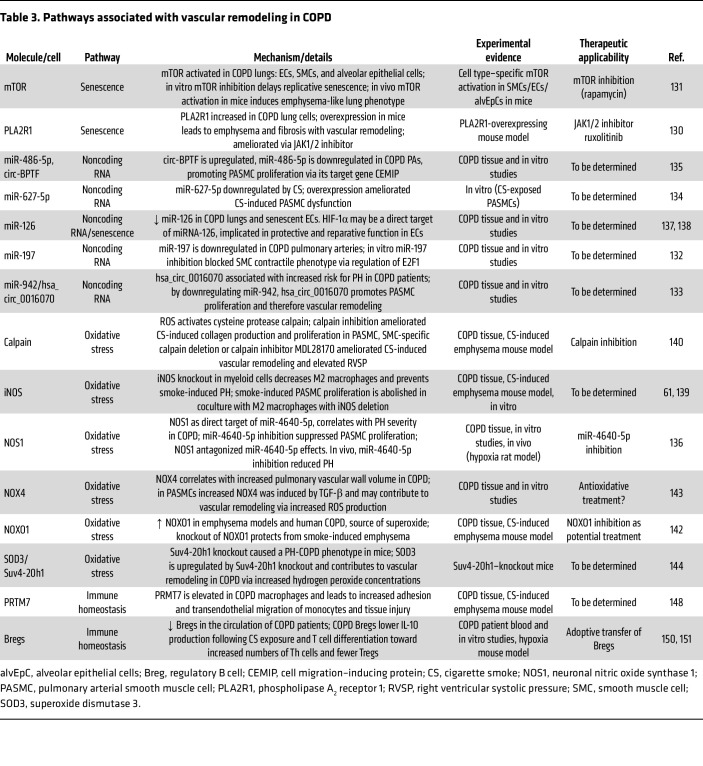
Pathways associated with vascular remodeling in COPD

**Table 4:**
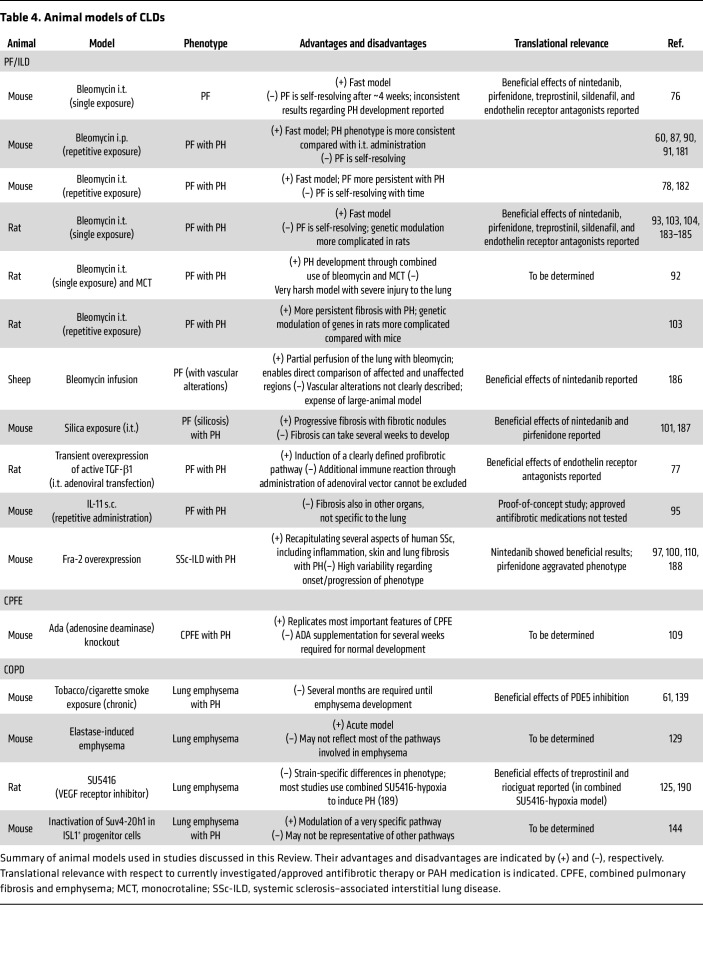
Animal models of CLDs

## Version 1 08/15/2023

Electronic publication
